# Editorial: Diet and exercise-induced inflammation

**DOI:** 10.3389/fnut.2024.1438832

**Published:** 2024-06-17

**Authors:** Maryam Akbari-Fakhrabadi, Mojtaba Kaviani, Diego Fernández-Lázaro

**Affiliations:** ^1^IWK Health Centre, Centre for Research in Family Health, Halifax, NS, Canada; ^2^School of Nutrition and Dietetics, Faculty of Pure and Applied Science, Acadia University, Wolfville, NS, Canada; ^3^Department of Cellular Biology, Genetic, Histology and Pharmacology, Faculty of Health Sciences, University of Valladolid, Campus de Soria, Soria, Spain; ^4^Neurobiology Research Group, Faculty of Medicine, University of Valladolid, Valladolid, Spain

**Keywords:** inflammation, diet, immune response, probiotics, endurance exercise

Post-exercise inflammatory response, characterized by increase in circulating inflammatory mediators produced by immune cells is a normal physiological process that is thought to play a vital role in tissue damage repair and enhancing muscle adaptation (1). However, physical exertion at a very high level of intensity for a prolonged time triggers the body to initiate a defense response through hormone release, the synthesis of acute phase proteins, and shifts in fluid and metabolic balance. Intense activities such as long-distance running promote the increase of inflammatory factors such as IL-6, IL-8, and C-reactive protein (CRP) which could influence an athlete's performance (2).

Diet plays a crucial role in providing energy and optimizing athletes' performance and recovery. The influence of diet in terms of inflammatory response in exercise is a vast area of study that has gained much attention in recent years. Potential dietary components could modulate exercise-induced cellular signals that stimulate inflammatory factors during intense physical activity (3). In this sense, Daily nutritional strategies that meet energy demands and provide sufficient specific macro- and micronutrients would support immune function (4). For instance, probiotics, prebiotics, or other functional foods are thought to have the potency to modify gut microbiota composition and improve the conditions of the intestinal epithelium and the immune system response to control inflammation, improve energy availability, and ultimately improve performance in athletes (5). The ongoing research in this field is giving us more insight into how different diets and specific dietary components could attenuate inflammatory biomarkers to benefit the overall performance of elite athletes (6).

In this Research Topic, *Diet and exercise-induced inflammation*, we aim to present the latest findings related to the interplay of nutrition and exercise-induced inflammation varying from research on cellular pathways to physical performance. We describe the most influential nutritional resources for modulated exercise-induced inflammation. Studies on proteins, amino acids, carbohydrates, antioxidants, and dietary supplements have demonstrated their importance and effectiveness in modulating acute and systemic inflammation. It is also essential to consider the guidelines on quantity, time, and composition of each of the nutritional elements to maximize their effectiveness, considering the principle of sports specificity.

This Research Topic of Frontiers in Nutrition, entitled “*Diet and exercise-induced inflammation*,” has gathered together six articles (Tan et al.; Przewłócka et al.; Morelli et al.; Khalafi et al.; Hurst, Lyall, Wells et al.; Hurst, Lyall, Roberts et al.): 4 original research (Przewłócka et al.; Morelli et al.; Hurst, Lyall, Wells et al.; Hurst, Lyall, Roberts et al.), 1 kinematic perspective study (Tan et al.), and 1 systematic review (Khalafi et al.).

Hurst et al., in two different study designs (Hurst, Lyall, Wells et al.; Hurst, Lyall, Roberts et al.) evaluated the effect of oral supplementation of New Zealand blackcurrant anthocyanin-rich extract (BAE) acutely 1 h before exercise (Hurst, Lyall, Roberts et al.) or chronically for 5 weeks (Hurst, Lyall, Wells et al.) on exercise-induced modulation of oxidative stress (Hurst, Lyall, Wells et al.; Hurst, Lyall, Roberts et al.), inflammation (Hurst, Lyall, Wells et al.; Hurst, Lyall, Roberts et al.), and circulating neutrophil function (Hurst, Lyall, Roberts et al.). The results from the acute intervention (Hurst, Lyall, Roberts et al.) revealed a time- and dose-dependent increase in plasma anthocyanins which peaked after 2 h of ingesting 3.2 mg/kg BAE supplementation. Supplementation with BAE (>1.6 mg/kg) 1 h before exercise facilitated recovery from exercise-induced oxidative stress with significant reductions in plasma oxidative capacity and post-exercise protein carbonyl levels compared to the placebo group. Furthermore, BAE preserved circulating neutrophil function (dose-dependent), because it attenuated the transient decrease in circulating neutrophils observed in the control group immediately after exercise. In the long-term intervention study, daily supplementation of 3.2 mg/kg BAE for 5 weeks improved post-exercise recovery due to its antioxidant and anti-inflammatory actions. In this sense, supplementation with BAE significantly reduced malondialdehyde (MDA) compared to the placebo group and increased plasma IL-10, salivary beta-defensin 2 (BD2), and secretory IgA (Hurst, Lyall, Wells et al.).

The results of the study conducted by Przewłócka et al. indicated that a 4-week supplementation of probiotics plus vitamin D3 substantially modifies alpha and beta diversity on the composition of gut microbiota in mixed martial arts (MMA) athletes. In the time-to-exhaustion exercise test, these researchers described improved epithelial cell permeability and improved athletic performance in MMA athletes (Przewłócka et al.). Strategies targeting the gut microbiome and amplified by the addition of vitamin D supplementation would improve suppression of intestinal inflammation through negative regulation of Toll-like receptor (TLR) expression (7), as well as enhanced innate immunity through different mechanisms, such as positive regulation of immunoglobulins, antimicrobial proteins, phagocytic activity, and natural killer cell activity, and improvements in T and B lymphocyte function (5).

The included perspective article (Tan et al.) in this Research Topic allows a better understanding of the nutritional mechanisms of bioflavonoids from a kinesiological perspective for their possible application in sports rehabilitation. Bioflavonoids have properties that modulate inflammation and reduce oxidative stress. These actions are due to the ability of bioflavonoids to reduce inflammation by inhibiting proinflammatory cytokines, such as tumor necrosis factor-alpha (TNF-α) and interleukin-6 (IL-6) (8). Furthermore, it also improves the activity of antioxidant enzymes and keeps reactive oxygen species (ROS) at low levels to prevent oxidative stress (9). This evidence would help reduce muscle damage and inflammation, thus speeding up the recovery process in athletes (Tan et al.).

Khalafi et al. in a systematic review reported that the increase in IL-15 is a transient response to exercise in trained and untrained individuals. IL-15 is a myokine with important metabolic, anabolic, and immune functions, therefore, the changes induced by exercise could be beneficial for the prevention and treatment of disorders in lipid and glucose metabolism (10), and pharmacologically beneficial in cancer immunotherapy through antitumor activity (11).

It has been previously described that adherence to the Mediterranean Diet and physical activity in adolescence represent powerful indicators of a healthy lifestyle in adulthood as a strategy for the prevention of chronic non-communicable diseases throughout life (12).

The study conducted by Morelli et al. in adolescents reported that a Mediterranean nutritional education Program (NEP) and/or physical activity after a 6-month intervention period improves adherence to the Mediterranean Diet. NEP negatively affected inflammatory biomarkers, ferritin, and CRP, whilst physical activity solely or combined with NEP had no impacts on inflammatory biomarkers (Morelli et al.).

The diversity of articles published in this Research Topic (Tan et al.; Przewłócka et al.; Morelli et al.; Khalafi et al.; Hurst, Lyall, Wells et al.; Hurst, Lyall, Roberts et al.) highlights the importance of healthy diets not only for optimal performance but also for overall health and recovery in active lifestyles. These studies shed light on the complex connections among dietary intake, inflammatory responses, and physical recovery.

By understanding these complex interrelations, we can develop periodized and personalized nutritional strategies aimed at attenuating systemic inflammation, accelerating recovery processes, and preventing chronic diseases. Thus, further research is warranted to provide comprehensive wellness initiatives, thereby supporting both elite athletes and the general population to optimize their health and fitness goals.

## Author contributions

MA-F: Data curation, Investigation, Supervision, Validation, Visualization, Writing – review & editing. MK: Data curation, Investigation, Supervision, Validation, Visualization, Writing – review & editing. DF-L: Conceptualization, Data curation, Formal analysis, Investigation, Validation, Visualization, Writing – original draft.
